# Lower adherence to healthy lifestyle is associated with increased adiposity and cardiovascular risk in youth

**DOI:** 10.3389/fnut.2026.1805649

**Published:** 2026-06-29

**Authors:** Antonietta Messina, Maria Casillo, Antonietta Monda, Girolamo Di Maio, Vincenzo Monda, Mohamed Salem Baazab, Marco La Marra, Marcellino Monda, Giovanni Messina, Fiorenzo Moscatelli, Rita Polito

**Affiliations:** 1 Department of Precision Medicine, University of Campania “Luigi Vanvitelli”, Naples, Italy; 2 Unit of Dietetics and Sports Medicine, Section of Human Physiology, Department of Experimental Medicine, University of Campania “Luigi Vanvitelli”, Naples, Italy; 3 Department of Human Science and Quality of Life Promotion, San Raffaele Telematic University, Rome, Italy; 4 Department of Psychology and Health Sciences, Pegaso Telematic University, Naples, Italy; 5 Department of Economics, Law, Cybersecurity, and Sports Sciences, University of Naples “Parthenope”, Naples, Italy; 6 Faculty of Medicine and Health Sciences, University of Aden, Aden, Yemen; 7 Department of Education and Sport Sciences, Pegaso Telematic University, Naples, Italy

**Keywords:** adiposity, blood pressure, body composition, cardiometabolic risk, lifestyle risk index, Mediterranean diet, pediatric obesity, physical activity

## Abstract

Unhealthy dietary patterns and insufficient physical activity are major contributors to pediatric overweight and obesity, yet their combined impact on body composition and blood pressure in youth is not fully understood. In this cross-sectional study, 24 participants (12 males, 12 females; 11–14 years) attending a pediatric lifestyle clinic were assessed. Adherence to the Mediterranean diet was evaluated with the MEDI-LITE questionnaire, and physical activity with the International Physical Activity Questionnaire (IPAQ); total physical activity was log-transformed. A composite Lifestyle Risk Index was constructed by standardizing MEDI-LITE scores and log-MET values (higher scores 38 = poorer diet and lower activity). Anthropometry, body mass index (BMI), waist circumference and body composition (fat mass percentage, FM%) were measured by standardized methods and multifrequency bioimpedance. Systolic and diastolic blood pressure (SBP, DBP) were measured at rest. Associations were examined using Spearman correlations, Mann–Whitney tests across lifestyle-risk strata, and multivariable linear regression adjusted for age and sex. Higher MEDI-LITE scores were associated with lower BMI and waist circumference, while higher physical activity was inversely associated with fat mass percentage and waist circumference. A composite Lifestyle Risk Index— reflecting lower adherence to the Mediterranean diet and lower physical activity levels—showed stronger associations with BMI, waist circumference, and fat mass percentage. Compared with the lower-risk group, the higher-risk group displayed higher adiposity measures. In regression models, the Lifestyle Risk Index remained associated with BMI, waist circumference, and fat mass percentage after adjustment for age and sex. No direct associations emerged between lifestyle variables and blood pressure, whereas BMI was positively associated with systolic blood pressure. These findings suggest that, in this exploratory pediatric sample, less favorable lifestyle patterns are associated with higher adiposity, which may partially explain variations in systolic blood pressure.

## 1 Introduction

In recent decades, profound changes in lifestyle behaviors have substantially influenced the health profile of pediatric and adolescent populations worldwide. Unhealthy dietary patterns and insufficient levels of physical activity have emerged as key determinants of the increasing prevalence of overweight and obesity already during childhood and adolescence. This trend represents a major public health concern, as excess body weight in early life is strongly associated with the premature development of cardiometabolic risk factors and with the persistence of obesity and related diseases into adulthood (1, 2). Accumulating evidence suggests that alterations in body composition and fat distribution may occur early, even before the onset of clinically overt metabolic disorders, highlighting the importance of early identification and prevention strategies (3).

Poor diet quality, characterized by high intake of energy-dense and nutrient-poor foods, together with sedentary behaviors, has been shown to adversely affect total and central adiposity, insulin sensitivity, lipid profile, and inflammatory status in young individual (4, 5). In particular, central fat accumulation has been recognized as a critical determinant of cardiometabolic risk, independently of overall BMI (6). Moreover, unfavorable lifestyle habits during growth may contribute to early vascular alterations and elevated blood pressure, setting the stage for future cardiovascular disease (7). Given the plasticity of metabolic and behavioral pathways during childhood and adolescence, this life stage represents a crucial window for preventive interventions aimed at reducing long-term health risks (8).

Dietary habits and physical activity are widely acknowledged as two of the most influential and modifiable components of lifestyle. However, growing evidence indicates that their effects on body composition and cardiometabolic health are not independent but rather interact in a complex and synergistic manner. Adherence to healthy dietary patterns, such as the Mediterranean diet, has been consistently associated with lower adiposity levels, improved body composition, and a more favorable metabolic profile in pediatric populations (9–11). Conversely, low adherence to the Mediterranean diet has been linked to increased BMI, higher waist circumference, and greater fat mass (12).

Similarly, insufficient physical activity and prolonged sedentary time have been associated with increased total and central adiposity, reduced lean mass, and poorer cardiometabolic outcomes in children and adolescents (13). Despite extensive evidence on diet and physical activity as independent determinants of pediatric health, their combined effect on body composition and cardiovascular risk remains insufficiently explored (14). In particular, few studies have integrated these lifestyle components into a composite index capable of capturing their synergistic influence in real-world settings (15, 16). Moreover, the extent to which lifestyle behaviors are directly associated with blood pressure, or indirectly through adiposity, is still unclear in pediatric populations. Addressing these gaps may improve the identification of early risk profiles and inform more effective preventive strategies (17, 18).

Importantly, only a limited number of studies have attempted to integrate dietary quality and physical activity into a single composite indicator of lifestyle risk, and even fewer have explored its association with multiple anthropometric parameters and blood pressure simultaneously (19). The use of composite lifestyle indices may allow for a more comprehensive assessment of behavioral risk profiles and their combined influence on body composition and cardiovascular markers. Moreover, evaluating whether adiposity mediates the relationship between lifestyle behaviors and blood pressure could provide valuable insights into the biological pathways underlying early cardiovascular alterations.

Against this background, the present study aimed to investigate the relationship between lifestyle behaviors and cardiometabolic-related anthropometric parameters in a pediatric and adolescent population, with particular emphasis on the combined contribution of dietary habits and physical activity levels (20, 21). Specifically, we sought to evaluate whether a lifestyle profile characterized by lower adherence to the Mediterranean diet and lower levels of physical activity is associated with adverse anthropometric outcomes, including BMI, body composition, and central adiposity. In addition, we examined the association between lifestyle behaviors and blood pressure values and explored whether any observed relationships with systolic or diastolic blood pressure are mediated by adiposity-related parameters (22). To address these aims, dietary adherence and physical activity were integrated into a composite lifestyle risk index, allowing for the assessment of their combined impact on anthropometric and hemodynamic outcomes (23). From a developmental perspective, blood pressure regulation during early adolescence is influenced by ongoing vascular, hormonal, and autonomic maturation. In this age range (11–14 years), increases in adiposity may contribute to early alterations in vascular function, sympathetic nervous system activity, and inflammatory pathways, potentially affecting systolic blood pressure even before clinically overt hypertension develops. These processes may partly explain why indirect associations between lifestyle and blood pressure, mediated by adiposity, are more detectable than direct relationships in pediatric populations. By adopting this integrated analytical approach, the study aims to contribute to a more comprehensive understanding of how modifiable lifestyle factors jointly influence body composition and cardiovascular risk markers at an early stage of life, thereby informing preventive strategies targeting pediatric and adolescent populations.

## 2 Materials and methods

### 2.1 Study design and participants

A total of 24 participants (12 males and 12 females), aged 11–14 years, were recruited from the Dietetics, Sports Medicine, and Psychophysical Well-being Unit of the University Hospital “Luigi Vanvitelli” (University of Campania), coordinated by Professor Marcellino Monda. This was a cross-sectional observational study conducted in a pediatric and adolescent population. Participants were consecutively recruited among adolescents attending the Dietetics, Sports Medicine, and Psychophysical Well-being Unit for clinical evaluation. Some participants may have been undergoing lifestyle counseling or follow-up, which could have influenced dietary habits and physical activity levels. All participants had complete data available for dietary habits, physical activity, anthropometric measurements, body composition, and blood pressure; therefore, a complete-case approach was applied for all analyses involving lifestyle variables. Inclusion criteria comprised availability of valid dietary and physical activity questionnaires and completion of anthropometric and clinical assessments. No specific exclusion criteria were applied other than missing data for key variables. Participants with missing data for key lifestyle variables were excluded from the analyses. Participants were recruited from a clinical setting, which may limit generalizability and introduce selection bias. Written informed consent was obtained from the parents or legal guardians of all participants, and assent was obtained from the adolescents prior to participation. The study was approved by the Ethics Committee of the University of Campania “Luigi Vanvitelli” (protocol 0013787/i, 23 April 2025).

### 2.2 Assessment of dietary adherence

Dietary habits were evaluated using the MEDI-LITE questionnaire, a validated tool designed to assess adherence to the Mediterranean diet. The questionnaire generates a total MEDI-LITE score, with higher values indicating greater adherence to Mediterranean dietary patterns. The total score was treated as a continuous variable for correlation and regression analyses (24).

### 2.3 Assessment of physical activity

Physical activity was assessed using the short form of the International Physical Activity Questionnaire (IPAQ-SF) (25). Total physical activity was calculated as MET-min·week^−1^, summing the contributions of walking, moderate-intensity, and vigorous-intensity activities according to standard IPAQ scoring procedures.

Given the marked positive skewness and presence of extreme values in total MET-min·week^−1^, physical activity data were log-transformed prior to inferential analyses using the following formula:


$$\log_{10}\;(\text{TOTAL}\;\mathrm{MET}+1)$$

This transformation was applied to improve normality and reduce the influence of outliers. Although widely used, the IPAQ has limitations in pediatric populations and may be affected by recall bias and overestimation of activity levels. Therefore, results should be interpreted with caution.

### 2.4 Construction of the lifestyle risk index

To capture the combined impact of diet quality and physical activity, a composite Lifestyle Risk Index was created. This index integrated the MEDI-LITE total score and log-transformed physical activity values, with higher index values reflecting a less favorable lifestyle profile, characterized by lower adherence to the Mediterranean diet and lower levels of physical activity. The Lifestyle Risk Index was treated as a continuous variable in correlation and regression analyses. For subgroup analyses, participants were further classified into lower-risk and higher-risk lifestyle groups based on the median value of the Lifestyle Risk Index.

The Lifestyle Risk Index was calculated as follows:


$$\text{Lifestyle Risk Index}=(-Z_{\text{MEDI}-\text{LITE}})+(-Z_{\log\;(\mathrm{MET}+1)})$$

where higher values indicate lower adherence to the Mediterranean diet and lower physical activity levels. The Lifestyle Risk Index has no fixed theoretical range, as it is based on standardized (z-score) variables. Both components (diet and physical activity) contributed equally to the index, as no weighting factors were applied.

### 2.5 Anthropometric and body composition measurements

- Weight and height were measured with standardized instruments.
- BMI was calculated as the weight/height ratio2 (kg/m^2^).
- Body composition by Bioimpedance Analysis (BIA): the assessment was performed with a multifrequency analyzer (BIA 101 Anniversary, Akern Srl, Florence, Italy) under standardized conditions (fasting for at least 8 h, empty bladder, and avoiding intense physical activity in the previous 24 h). BIA allows for the estimation of fat mass, lean mass, total body water, and intracellular and extracellular compartments, providing a detailed picture of body composition.

Systolic and diastolic blood pressure were measured under standardized resting conditions using an appropriate cuff size. Blood pressure values were treated as continuous variables in all analyses.

The bioimpedance analysis device uses predictive equations that have been previously applied in adolescent populations; however, estimations may be influenced by hydration status and should be interpreted with caution.

### 2.6 Statistical analysis

All statistical analyses were conducted on the final study sample comprising 24 participants with complete data for dietary adherence and physical activity. Continuous variables were inspected for distributional properties through visual examination of histograms and Q–Q plots. Physical activity data derived from the International Physical Activity Questionnaire (IPAQ) exhibited marked positive skewness and the presence of extreme values; therefore, total physical activity (MET-min·week^−1^) was log-transformed using the formula log_10_ (TOTAL MET + 1) prior to inferential analyses.

Dietary adherence was assessed using the MEDI-LITE total score, with higher values indicating greater adherence to the Mediterranean diet. To evaluate the combined contribution of diet quality and physical activity, a composite Lifestyle Risk Index was constructed. Specifically, MEDI-LITE total scores and log-transformed total MET values were first standardized (z-scores). The standardized physical activity component was sign-inverted so that higher values reflected lower activity levels. The Lifestyle Risk Index was then calculated as the sum of the standardized poor-diet and low-activity components, with higher index values indicating a less favorable lifestyle profile.

Associations between lifestyle-related variables and anthropometric parameters were assessed using Spearman’s rank correlation coefficient, given the ordinal nature of questionnaire-derived scores and the non-normal distribution of several variables. Correlation analyses were performed for BMI, waist circumference, and fat mass percentage (FM%).

Participants were further categorized into low-risk and high-risk lifestyle groups based on the median value of the Lifestyle Risk Index. Between-group comparisons of anthropometric variables were conducted using the Mann–Whitney U test. Effect sizes for between-group differences were quantified using rank-biserial correlation, providing an estimate of the magnitude of group separation independent of sample size.

To examine independent associations between lifestyle behaviors and anthropometric outcomes, multivariable linear regression models were fitted with BMI, waist circumference, and FM% as dependent variables. The Lifestyle Risk Index was entered as the main predictor, and all models were adjusted for age and sex. Regression coefficients (*β*), 95% confidence intervals (CI), and *p*-values are reported.

Additional regression analyses were conducted to explore the relationship between adiposity and blood pressure. Systolic and diastolic blood pressure were analyzed in separate models with BMI as the primary predictor, adjusting for age and sex. Statistical significance was set at *p* < 0.05 for all analyses.

All statistical analyses were performed using IBM SPSS Statistics for Windows, Version 28.0 (IBM Corp., Armonk, NY, USA). Given the relatively small sample size, regression analyses were considered exploratory, and results should be interpreted with caution, particularly with regard to potential overfitting.

## 3 Results

### 3.1 Participant characteristics and data preparation

A total of 24 participants were included in the study. Based on age- and sex-specific pediatric BMI cut-offs, 8 adolescents were classified as having overweight and 16 as having obesity. Thus, all participants presented excess body weight, confirming the clinical profile of the study sample. Complete data for dietary adherence (MEDI-LITE total score) and physical activity (IPAQ total MET-min·week^−1^) were available for 24 participants; therefore, analyses involving lifestyle variables were conducted on this complete-case subset. Physical activity data derived from the IPAQ exhibited marked positive skewness and the presence of extreme values; consequently, total physical activity was log-transformed as log_10_ (TOTAL MET + 1) prior to inferential analyses. To evaluate the combined impact of diet quality and physical activity, a composite Lifestyle Risk Index was constructed, with higher values reflecting poorer dietary adherence and lower physical activity levels.

### 3.2 Associations between lifestyle behaviors and anthropometric parameters

**Figure 1:**
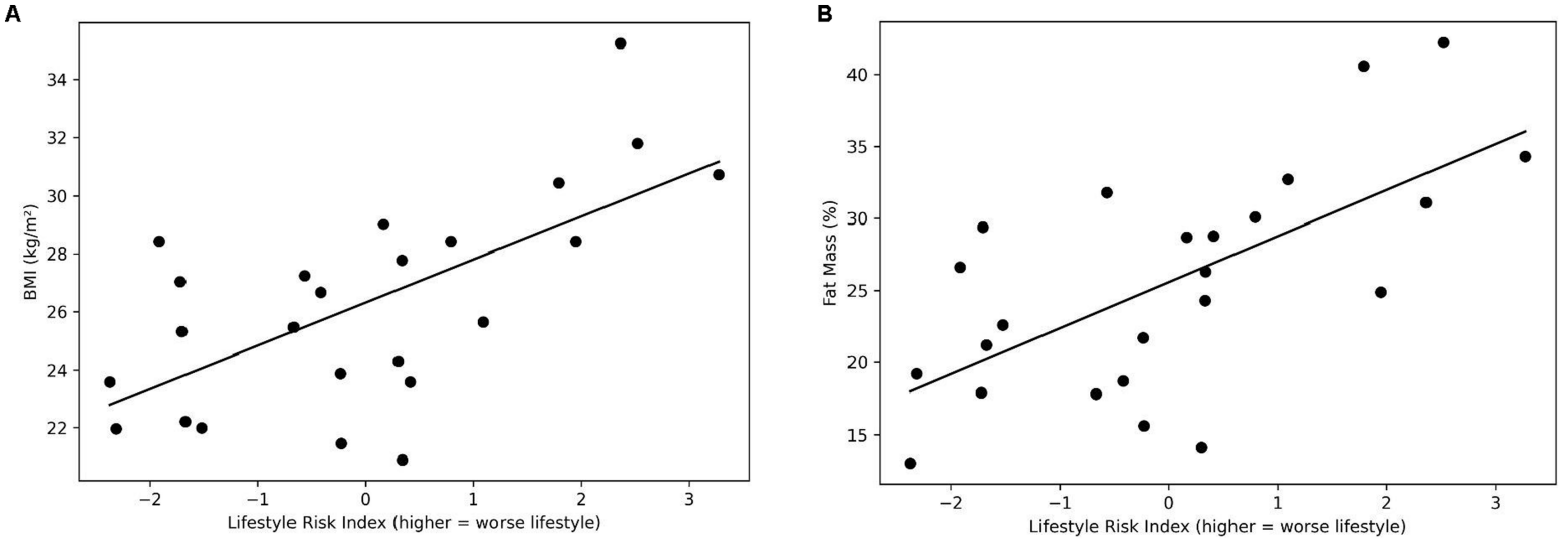
**(A)** Lifestyle risk index and BMI. Scatter plot illustrating the relationship between the Lifestyle Risk Index and BMI. The Lifestyle Risk Index integrates lower adherence to the Mediterranean diet (MEDI-LITE total score) and lower levels of physical activity (log-transformed total MET-min·week-1), with higher values indicating a less favorable lifestyle profile. The association was assessed using Spearman’s rank correlation coefficient, showing that higher Lifestyle Risk Index values were significantly associated with higher BMI, reflecting a worse anthropometric profile among participants with poorer lifestyle behaviors. **(B)** Lifestyle risk and fat mass percentage. Scatter plot depicting the relationship between the Lifestyle Risk Index and fat mass percentage (FM%). The Lifestyle Risk Index integrates lower adherence to the Mediterranean diet (MEDI-LITE total score) and lower levels of physical activity (log-transformed total MET-min·week-1), with higher values indicating a poorer lifestyle profile. The association was evaluated using Spearman’s rank correlation coefficient. Higher Lifestyle Risk Index values were significantly associated with increased fat mass percentage, indicating greater adiposity among participants with less healthy lifestyle behaviors.

Bivariate analyses revealed consistent associations between lifestyle behaviors and anthropometric measures. Higher adherence to the Mediterranean diet, as reflected by a higher MEDI-LITE total score, was significantly associated with lower BMI (Spearman’s *ρ* = −0.62, *p* = 0.001) and smaller waist circumference (*ρ* = −0.54, *p* = 0.006). Similarly, higher physical activity levels, assessed as log-transformed total MET-min·week^−1^, were inversely associated with fat mass percentage (FM%; *ρ* = −0.60, *p* = 0.002) and waist circumference (*ρ* = −0.41, *p* = 0.048). When diet and physical activity were combined into the Lifestyle Risk Index, stronger and more consistent associations with anthropometric outcomes emerged. Higher Lifestyle Risk Index values were positively correlated with BMI (*ρ* = 0.54, *p* = 0.006), waist circumference (*ρ* = 0.53, *p* = 0.008), and FM% (*ρ* = 0.64, *p* < 0.001), indicating that participants characterized by poorer diet and lower physical activity exhibited a markedly worse body composition profile (Figures 1A,B).

### 3.3 Comparison across lifestyle risk strata

**Figure 2:**
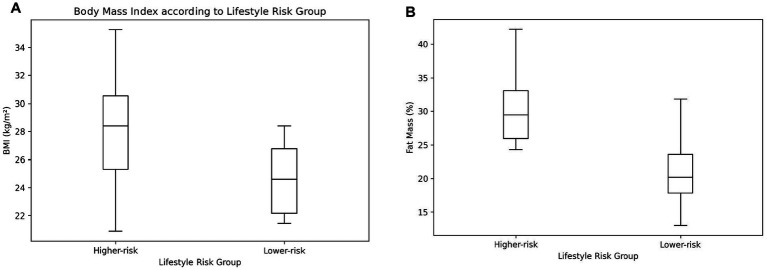
**(A)** BMI according to lifestyle risk group. Boxplot illustrating BMI stratified by Lifestyle Risk Group (low risk vs. high risk), defined according to the median value of the Lifestyle Risk Index. The Lifestyle Risk Index reflects the combined effect of lower adherence to the Mediterranean diet and lower levels of physical activity. Participants classified in the high lifestyle risk group exhibited significantly higher BMI compared with those in the low-risk group, indicating a less favorable anthropometric profile associated with poorer lifestyle behaviors. **(B)** Fat mass percentage according to lifestyle risk group. Boxplot illustrating fat mass percentage (FM%) stratified by Lifestyle Risk Group (low risk vs. high risk), defined according to the median value of the Lifestyle Risk Index. The Lifestyle Risk Index reflects the combined effect of lower adherence to the Mediterranean diet and lower levels of physical activity. Participants in the high lifestyle risk group exhibited significantly higher fat mass percentage compared with those in the low-risk group, indicating greater adiposity associated with a less favorable lifestyle profile.

Participants were further stratified into higher-risk and lower-risk lifestyle groups based on the median value of the Lifestyle Risk Index. Individuals in the higher-risk group showed significantly higher BMI (28.0 vs. 24.6 kg·m^−2^, *p* = 0.022), larger waist circumference (92.7 vs. 80.2 cm, *p* = 0.024), and higher FM% (29.8 vs. 21.3%, *p* = 0.009) compared with those in the lower-risk group. Effect size estimates indicated large between-group differences for all anthropometric outcomes (Figures 2A,B), supporting the presence of clinically meaningful disparities associated with lifestyle behaviors. The observed differences between lifestyle risk groups are not only statistically significant but may also be clinically meaningful, particularly given the magnitude of differences in adiposity-related parameters during a critical developmental stage.

### 3.4 Multivariable analyses

**Figure 3:**
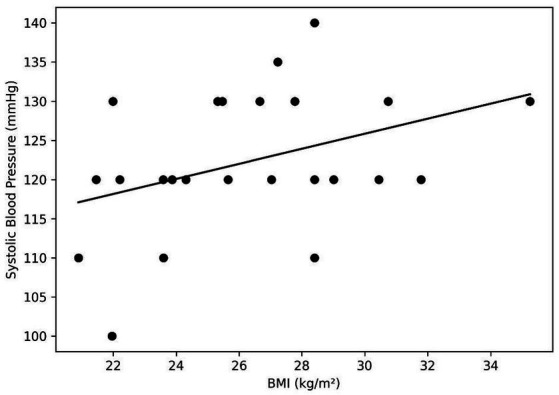
Association between BMI and systolic blood pressure. Scatter plot illustrating the relationship between BMI and systolic blood pressure (SBP). Each point represents an individual participant. The solid line represents the fitted linear trend, indicating a positive association between BMI and systolic blood pressure. This finding supports the presence of higher systolic blood pressure values with increasing adiposity, in line with the regression analyses adjusted for age and sex reported in the Results.

In multivariable linear regression models adjusted for age and sex, the Lifestyle Risk Index remained a significant independent predictor of adverse anthropometric parameters. Specifically, each one-unit increase in the Lifestyle Risk Index was associated with a 1.24 kg·m^−2^ increase in BMI (95% CI: 0.37–2.11, *p* = 0.005), a 2.70 cm increase in waist circumference (95% CI: 0.20 to 5.20, *p* = 0.034), and a 3.62% increase in FM% (95% CI: 2.26–4.97, *p* < 0.001). These findings indicate that the combined effect of poorer diet quality and reduced physical activity is independently associated with increased overall and central adiposity. Direct associations between lifestyle variables and blood pressure were weaker. Neither the MEDI-LITE total score nor physical activity levels, alone or combined within the Lifestyle Risk Index, were significantly associated with systolic or diastolic blood pressure in bivariate or multivariable analyses. However, adiposity-related parameters showed relevant associations with blood pressure: BMI was positively associated with systolic blood pressure in age- and sex-adjusted models (*β* = 1.35 mmHg per 1 kg·m^−2^ increase in BMI; 95% CI: 0.17–2.54; *p* = 0.025). No significant association was observed between BMI and diastolic blood pressure (*p* > 0.05). This pattern suggests that the relationship between lifestyle behaviors and blood pressure may be predominantly mediated by body composition rather than reflecting a direct effect of diet or physical activity on resting blood pressure levels (Figure 3).

## 4 Discussion

The present study investigated the relationship between lifestyle behaviors and anthropometric and hemodynamic parameters in a pediatric and adolescent population, with a specific focus on the combined contribution of dietary habits and physical activity levels. The main finding is that a lifestyle profile characterized by lower adherence to the Mediterranean diet and reduced physical activity is associated with a less favorable anthropometric profile, including higher BMI, greater total fat mass, and increased central adiposity. In contrast, no direct association emerged between lifestyle behaviors and blood pressure values; however, adiposity—particularly BMI—was positively associated with systolic blood pressure, suggesting that body composition may mediate the relationship between lifestyle and blood pressure at this stage of life.

The associations observed between lifestyle behaviors and anthropometric parameters are biologically plausible and consistent with current knowledge. Higher adherence to the Mediterranean diet has been widely associated with improved body weight regulation, largely due to better control of energy intake, higher dietary fiber consumption, favorable fatty acid profiles, and reduced intake of ultra-processed foods (26, 27). These dietary characteristics contribute to improved satiety, insulin sensitivity, and metabolic efficiency, which may collectively limit excessive fat accumulation.

Similarly, physical activity plays a fundamental role in maintaining energy balance and regulating body composition, particularly by preserving fat-free mass and reducing fat mass (28). In the present study, higher physical activity levels were associated with lower fat mass percentage and smaller waist circumference, reinforcing the notion that movement-related behaviors are especially relevant for adiposity distribution. Importantly, when diet quality and physical activity were integrated into a composite Lifestyle Risk Index, the associations with anthropometric outcomes became stronger and more consistent across analytical approaches. This finding supports the concept that lifestyle behaviors act synergistically rather than independently and that their combined assessment better captures real-world behavioral patterns (29).

The consistency of findings across different analytical approaches supports the robustness of the observed patterns, although confirmation in larger samples is needed. Associations were confirmed through bivariate correlations, comparisons between lifestyle risk groups, and multivariable regression models adjusted for age and sex. This convergence across different analytical strategies strengthens confidence in the robustness of the observed relationships and highlights the value of integrated lifestyle indicators in pediatric research (30).

The relationship between lifestyle behaviors and blood pressure requires a cautious and nuanced interpretation. In the present study, neither dietary adherence nor physical activity—considered individually or jointly within the Lifestyle Risk Index—showed a significant direct association with systolic or diastolic blood pressure. Conversely, BMI was positively associated with systolic blood pressure, suggesting that body size and adiposity-related status may be more closely related to systolic blood pressure than lifestyle variables in this sample. However, this interpretation should be considered cautiously, as BMI is an indirect proxy of adiposity and does not directly quantify fat mass or fat distribution (31).

These findings suggest that the impact of lifestyle behaviors on blood pressure may occur predominantly through indirect pathways mediated by body composition, rather than through immediate hemodynamic effects. This interpretation aligns with physiological models in which excess adiposity contributes to increased sympathetic activity, altered vascular function, and low-grade inflammation, all of which can influence systolic blood pressure. In pediatric and adolescent populations, however, these mechanisms may not yet be fully established, potentially explaining the absence of a direct association between lifestyle behaviors and blood pressure observed in this study.

Previous research in young populations has reported mixed findings regarding lifestyle–blood pressure associations, with stronger and more consistent relationships typically emerging in adulthood (32). The present results therefore support the notion that early lifestyle behaviors primarily influence cardiovascular risk indirectly by shaping body composition, while direct effects on blood pressure may require longer exposure or the presence of more advanced metabolic alterations.

Several strengths enhance the relevance of this study. First, the integration of dietary adherence and physical activity into a single Lifestyle Risk Index represents an innovative and conceptually sound approach that reflects the multidimensional nature of lifestyle. Second, the use of objective anthropometric measures and multivariable analyses improves the reliability of the findings. Third, the cautious interpretation of blood pressure results avoids overstatement and adheres to the limits imposed by the data (17, 33).

This study has several limitations. The small sample size increases the risk of type I error and limits statistical power, particularly for multivariable analyses. The use of regression models in a small sample raises the possibility of overfitting. The clinical recruitment setting may introduce selection bias and limit generalizability (34). Physical activity was assessed using the IPAQ, which has known limitations in pediatric populations. Body composition was measured using bioimpedance analysis, which may be influenced by hydration status. Additionally, the lack of pubertal status assessment represents a potential confounding factor. Finally, the cross-sectional design precludes causal inference (35).

Overall, the present study emphasizes the importance of considering diet and physical activity together when evaluating lifestyle-related health risks in pediatric and adolescent populations. The findings suggest that adiposity is a central node linking lifestyle behaviors to early cardiovascular risk, particularly systolic blood pressure (31, 36). These results reinforce the need for early, integrated preventive strategies that simultaneously promote healthy eating patterns and regular physical activity to prevent excess adiposity and its downstream consequences.

Alternative explanations should also be considered. First, the cross-sectional design precludes causal inference, and reverse causality cannot be excluded, as higher adiposity may influence lifestyle behaviors. Second, the clinical nature of the sample may have amplified associations due to a higher baseline risk profile. Third, the lack of pubertal status assessment represents a relevant limitation, as hormonal and developmental factors may influence both adiposity and blood pressure in this age group. The main contribution of this study lies in the integrated evaluation of dietary and physical activity behaviors through a composite index, highlighting their combined association with adiposity-related outcomes in a pediatric clinical sample.

Our findings are consistent with previous studies showing that both dietary quality and physical activity are associated with adiposity-related outcomes in pediatric populations. In particular, adherence to the Mediterranean diet has been linked to lower BMI and improved cardiometabolic profiles in children and adolescents (12, 13, 26, 27), while higher physical activity levels have been associated with lower fat mass, reduced central adiposity, and more favorable cardiometabolic outcomes (11, 28, 29). However, consistent with previous pediatric studies reporting heterogeneous associations between lifestyle behaviors and blood pressure (16, 22, 33), we did not observe a direct association between lifestyle behaviors and blood pressure, supporting the hypothesis that BMI and adiposity-related parameters may represent intermediate factors linking lifestyle behaviors to early cardiovascular risk (34, 35). These findings contribute to the existing literature by highlighting the value of a combined lifestyle index in capturing the synergistic effects of behavioral factors. Nevertheless, future longitudinal studies involving larger cohorts are warranted to clarify causal pathways and to determine whether improvements in lifestyle behaviors during childhood and adolescence translate into sustained benefits for body composition and cardiovascular health later in life.

## Data Availability

The raw data supporting the conclusions of this article will be made available by the authors, without undue reservation.
